# Marine fog inputs appear to increase methylmercury bioaccumulation in a coastal terrestrial food web

**DOI:** 10.1038/s41598-019-54056-7

**Published:** 2019-11-26

**Authors:** Peter S. Weiss-Penzias, Michael S. Bank, Deana L. Clifford, Alicia Torregrosa, Belle Zheng, Wendy Lin, Christopher C. Wilmers

**Affiliations:** 1 Department of Microbiology and Environmental Toxicology, University of California, Santa Cruz, CA USA; 2 Institute of Marine Research, Department of Contaminants and Biohazards, Bergen, Norway; 3 University of Massachusetts, Department of Environmental Conservation, Amherst, MA USA; 4 Wildlife Investigations Lab, California Department of Fish and Wildlife, Rancho Cordova, CA USA; 5 University of California, School of Veterinary Medicine, Department of Medicine and Epidemiology, Davis, CA USA; 6 United States Geological Survey, Western Geographic Science Center, Menlo Park, CA USA; 7 Environmental Studies Department, University of California, Santa Cruz, CA USA

**Keywords:** Element cycles, Element cycles

## Abstract

Coastal marine atmospheric fog has recently been implicated as a potential source of ocean-derived monomethylmercury (MMHg) to coastal terrestrial ecosystems through the process of sea-to-land advection of foggy air masses followed by wet deposition. This study examined whether pumas (*Puma concolor*) in coastal central California, USA, and their associated food web, have elevated concentrations of MMHg, which could be indicative of their habitat being in a region that is regularly inundated with marine fog. We found that adult puma fur and fur-normalized whiskers in our marine fog-influenced study region had a mean (±SE) total Hg (THg) (a convenient surrogate for MMHg) concentration of 1544 ± 151 ng g^−1^ (N = 94), which was three times higher (*P* < 0.01) than mean THg in comparable samples from inland areas of California (492 ± 119 ng g^−1^, N = 18). Pumas in California eat primarily black-tailed and/or mule deer (*Odocoileus hemionus*), and THg in deer fur from the two regions was also significantly different (coastal 28.1 ± 2.9, N = 55, vs. inland 15.5 ± 1.5 ng g^−1^, N = 40). We suggest that atmospheric deposition of MMHg through fog may be contributing to this pattern, as we also observed significantly higher MMHg concentrations in lace lichen (*Ramalina menziesii*), a deer food and a bioindicator of atmospheric deposition, at sites with the highest fog frequencies. At these ocean-facing sites, deer samples had significantly higher THg concentrations compared to those from more inland bay-facing sites. Our results suggest that fog-borne MMHg, while likely a small fraction of Hg in all atmospheric deposition, may contribute, disproportionately, to the bioaccumulation of Hg to levels that approach toxicological thresholds in at least one apex predator. As global mercury levels increase, coastal food webs may be at risk to the toxicological effects of increased methylmercury burdens.

## Introduction

Mercury (Hg) is a globally distributed and ubiquitous pollutant found in terrestrial, freshwater and marine ecosystems^1,2^. The health threats to exposure are significant for ecosystems and human welfare worldwide^3^, but quantifying these risks is complicated by the many facets of mercury cycling which are inherently complex^4^ especially considering the effects of global change^5^. Unlike other metals, the atmosphere is the main pathway for Hg to be transported from sources (both natural and anthropogenic) to receptor regions^1,3^. With a global atmospheric lifetime against oxidation of 6–12 months^1,6^, gaseous elemental Hg (Hg°) is the dominant atmospheric Hg species and is hemispherically well-mixed. The primary sink for atmospheric Hg° is thought to be oxidation of Hg° to highly reactive and water soluble Hg^II^ compounds with atmospheric lifetimes on the order of days to weeks, removed through wet and dry deposition^7^. Hg° in the atmosphere also undergoes bidirectional air-surface exchange with soils and vegetation, which in the case of Hg-enriched soils results in a net source to the atmosphere^8^, and in the case of Arctic tundra vegetation and some grasslands results in net uptake from the atmosphere^9,10^. Organic Hg compounds (primarily monomethylmercury (MMHg)) are generally present in only trace amounts in the atmosphere^11–13^, as they are formed primarily in anaerobic soils, sediments, and waters, through the metabolic processes of a diverse array of microbes^14^. However, new research on atmospheric marine fog water deposition indicates a novel pathway through which coastal terrestrial food webs may be exposed to mercury^15,16^. Understanding this connection is particularly important since it is recognized that global environmental change can modulate the mercury risk to wildlife and humans through increased emissions, deposition and subsequent uptake by biota^4,5^.

In coastal zones with consistent periods of onshore advection of marine fog there can be significant wet deposition of fog drip^17–22^. An early study of total Hg in marine fog water in eastern Canada showed that fog water can account for as much as 31–74% of all Hg in wet deposition^23^. On the central coast of California, USA, coastal marine fog varies seasonally, partially overlapping in time with coastal upwelling in the California Current. Upwelling brings elevated concentrations of methylated Hg compounds to the surface waters from deeper, low-oxygen waters and/or sediments (where anaerobic microbes are active), whereby sea-air exchange of methylated Hg compounds can be source of gaseous dimethylmercury (DMHg) and/or gaseous and particulate MMHg to the atmosphere^24,25^. Dimethyl Hg in the atmosphere is unstable and will break down into MMHg in the gas phase^26^ or the aqueous phase at low pH^27^, followed by rapid adsorption onto aerosols and cloud droplets in the marine boundary layer^28^. Once MMHg is in the aqueous phase it may be stable for several days especially in clouds where sunlight is attenuated^29^. This marine based process results in elevated concentrations of MMHg in coastal fog drip^15,16^. The presence of MMHg in coastal cloud water is indicative of the air mass having had a recent source of ocean-derived methylated mercury^28^. This behavior is distinct from that of inorganic Hg compounds (Hg^II^), which can have both oceanic and land sources, and are generally not useful tracers of ocean emissions^28^.

Bioaccumulation of MMHg to top predators from aquatic sources is well documented^30–33^, which is largely due to the prevalence of anaerobic microbes in aquatic systems, plus the numerous food web positions that allow for efficient trophic transfer of MMHg. For terrestrial predators within food webs of terrestrial origins, the risk is thought to be lower, with the exception of sites close to highly polluted active mines^34^ and runoff from legacy mining activity^35^.

Lichens have been used successfully as a bioindicator of atmospheric pollutants, including mercury, by collecting individual specimens and measuring the pollutant concentrations in the thallus (plant tissue that lacks roots and a vascular system)^36–40^. Earlier work^41^ on lichen transplanted into a relatively polluted fog-laden environment showed a rapid uptake of atmospheric Hg with increases in total Hg concentration by nearly a factor of 2 after 12 months. A more recent study in the Arctic demonstrated that MMHg in lichen from the coast was highly enriched relative to the underlying soil, and this was indicative of atmospheric deposition from an oceanic source of organic Hg compounds^42^. These authors suggest that MMHg accumulation in lichen and caribou can potentially affect the health of the people who depend on caribou as part of a traditional diet.

The primary objective of this study was to evaluate whether ocean-derived MMHg from marine fog wet deposition to coastal landscapes could influence the Hg bioaccumulation patterns in coastal terrestrial food webs. Specifically, we used a multiple lines of evidence approach, across different trophic levels, to test whether biotic ecosystem compartments including pumas, their primary prey, mule deer (*Odocoileus hemionus*) and lace lichen (*Ramalina menziesii*), a deer food in temperate forests^43^ were each elevated in THg (for deer and puma) and MMHg (for lichen) at fog influenced coastal sites compared to inland sites with no fog from marine sources. Finally, we compared puma Hg concentrations to established mammalian toxicological thresholds.

## Results and Discussion

### Mercury concentrations in lichen, deer, and puma along a coastal fog gradient

**Figure 1:**
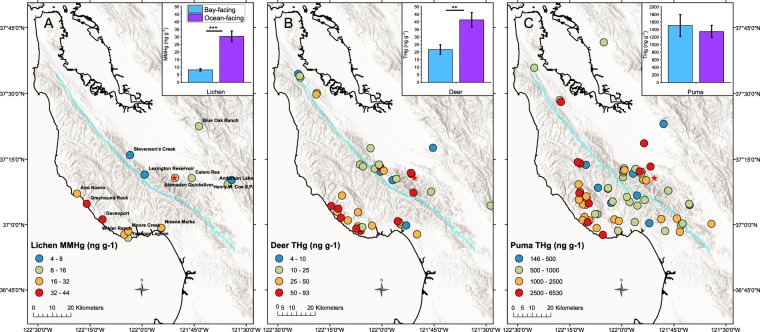
Geographical distributions in the Santa Cruz Mountain coastal region, California, USA, and mean (±1 SE) values by coastal sub-region of (**A**) MMHg concentrations in lace lichen (*Ramalina menziesii*) (site names correspond to data in Table S1), (**B**) THg in adult deer fur, and (**C**) THg in adult puma fur and fur-normalized whisker samples. The blue line represents the watershed boundary which was used to delineate samples as belonging to either the ocean facing (to the left of the line) or bay facing (to the right of the line) sub-regions. Asterisks indicate the p-value from one-way ANOVA test on the log-transformed concentrations, shown by the extent of the horizontal lines (**p = 0.001–0.01, ***p = <0.001). Sampling for lichen, deer and puma was done in 2017, 2008–2012, and 2006–2014, respectively.

Previous research has identified that the Santa Cruz Mountains in our study area, form an effective barrier to the inland penetration of marine fog^19^. Lichen, deer, and puma samples were taken on both sides of the watershed divide of this range, thus providing an opportunity to test the effects of varying summertime fog and low cloud cover (FLCC) on the observed MMHg and THg concentrations in biotic samples. The ocean-facing sites from which lichen samples were taken, located to the southwest of the watershed divide (Fig. 1A), averaged 6.2 hrs/day of summertime FLCC with a high of 9.5 hrs/day (Table S1, Supporting Information). Ocean-facing samples were <1.1 km from the Pacific Ocean whereas the bay-facing group were more spread out, located >24 km from the coast, and averaged 3.7 hrs/day summertime FLCC (Table S1). However, while this FLCC frequency is only 60% less than that for the ocean-facing sites, the difference in summertime fog wet deposition is likely to be at least an order of magnitude, with ocean-facing sites being wetter^21,22^.

The mean (±1 SE) MMHg concentration in lichen from ocean-facing sites was 30.2 ± 3.5 ng g^−1^, which is a 3.7 times enrichment (difference significant, ANOVA) over the mean from the bay-facing sites (8.2 ± 0.8 ng g^−1^) (Table 1, Figs. 1A and S1). This enrichment is comparable to that observed in lichen on Bathurst Island in the Canadian Arctic, where St. Pierre *et al*.^39^ observed a five times enrichment in lichen MMHg sampled on the coast nearest to ice leads exposing the open-ocean where DMHg emissions are known to occur^40^, compared to locations >20 km inland.

**Table 1:** Statistics on the subsets of concentration data used in this paper.

Dataset	N total	Mean	Standard Deviation	Standard Error	Minimum	Median	Maximum
coastal adult puma THg	94	1544	1462	150.8	145.9	1026	22052^*^
inland adult puma THg	18	491.8	503.1	118.6	72.0	304.9	1946
museum adult puma THg	9	289.7	125.0	41.7	114.8	266.4	524.0
coastal subadult puma THg	12	537.9	339.7	98.1	141.1	473.8	3092^*^
coastal kitten puma THg	16	384.3	167.0	41.7	75.8	400.6	4495^*^
inland subadult puma THg	6	205.6	96.5	39.4	99.4	199.3	337.7
inland kitten puma THg	5	69.3	38.9	17.4	26.8	59.4	132.9
bay-facing adult puma THg	27	1503	1501	288.9	145.9	867.1	6405
ocean-facing adult puma THg	53	1346	1159	159.2	237.9	965.5	6530
coastal adult male puma THg	41	1358	1043	162.9	237.9	1066	5399
coastal adult female puma THg	34	1626	1733	297.3	215.5	918.5	8079
inland adult male puma THg	11	510.9	411.0	123.9	72.0	484.5	1574
inland adult female puma THg	7	461.8	658.7	249.0	122.3	207.0	1946
inland deer THg	40	15.5	9.6	1.5	2.8	13.4	51.8
coastal deer THg	55	28.1	21.7	2.9	2.8	21.5	186.3^*^
ocean-facing deer THg	18	41.2	20.9	4.9	8.0	34.1	82.5
bay-facing deer THg	37	21.7	19.3	3.2	2.8	14.2	186.3^*^
ocean lichen MMHg	15	30.3	13.5	3.5	14.3	27.5	65.2
bay facing lichen MMHg	14	8.2	3.0	0.8	3.3	8.5	12.9
ocean-facing lichen THg	15	138.1	44.4	11.5	49.5	141.4	222.4
bay-facing lichen THg all	14	333.6	345.1	92.2	85.7	222.4	1354^*^
bay-facing lichen THg w/o Almaden	12	206.3	71.4	20.6	85.7	200.7	308.4

All concentrations are in ng g^−1^ (ppb). Concentrations denoted with an asterisk were considered outliers according to a Grubb’s test and were not included in the calculation of the statistical parameters. Sampling for lichen, deer and puma was done in 2017, 2008–2012, and 2006–2014, respectively, at locations in California, USA.

The mean THg concentration in central California bay-facing lichen was higher than that from ocean-facing lichen (Table 1), although the difference was not significant (ANOVA). Very high concentrations of THg were observed in lichen samples at one bay-facing site, Almaden Quicksilver, a former Hg mine (1097 ± 58.2 ng g^−1^), with levels that are close to an order of magnitude higher than THg in lichen from other locations (Table S1). However, in spite of the elevated THg concentrations, lichen samples from Almaden Quicksilver did not have enhanced MMHg concentrations. The mean MMHg in lichen from this site was 8.2 ± 3.3 ng g^−1^, the same value as the mean for all sites in the bay facing region. Overall, the lichen mean %MMHg (MMHg/THg × 100) in the ocean-facing region was 23.0% compared to 4.4% for the bay-facing region (N = 7), with the data from Almaden Quicksilver removed as an outlier (Table S1).

Samples of adult mule deer fur were collected in close proximity to the lichen sampling sites, in both ocean-facing and bay-facing sub-regions, and were analyzed for THg concentrations. These data are shown in Fig. 1B, and reveal that the mean THg concentration in deer samples from the ocean-facing region was significantly higher (41.2 ± 4.9 ng g^−1^, N = 18) than that from the bay-facing region (21.6 ± 3.2 ng g^−1^, N = 38, one outlier sample removed, see Tables 1 and S2).

Adult puma fur and whiskers sampled across the ocean- and bay-facing regions were also analyzed for THg (whisker THg concentrations are reported as fur-normalized concentrations, see Fig. S2). Mean THg concentrations between the two sub-regions were not significantly different (Fig. 1C) (ocean facing: 1346 ± 159 ng g^−1^, N = 53, bay facing: 1503 ± 299 ng g^−1^, N = 53, with one outlier removed, see Table 1 and Table S3). This lack of observed spatial pattern was expected due to the large home range size exhibited by adult pumas^44^.

### THg in coastal vs. inland deer and puma

**Figure 2:**
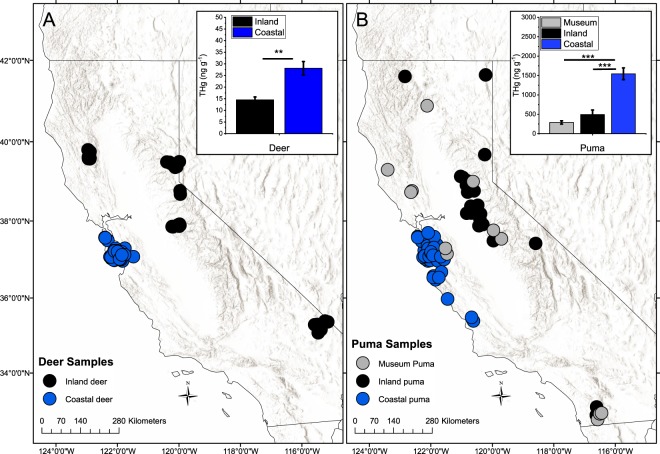
(**A**) Deer and (**B**) puma sample locations in California, USA, and mean (±1 SE) concentrations of total mercury (THg) in samples from each sub-region. Asterisks indicate the p-value from one-way ANOVA test on the log-transformed concentrations, shown by the extent of the horizontal lines (**p = 0.001–0.01, ***p = < 0.001). Sampling for deer and puma was done in 2008–2012, and 2006–2014, respectively.

A broader spatial comparison was made between THg concentrations in pumas and mule deer from the coastal region (which includes both the ocean-and bay-facing sub-regions) and the inland region of California (hundreds of km distant), which includes mountainous regions of the Klamath, Sierra Nevada and Cascade ranges, as well as desert regions in the southern part of the state. For mule deer, mean THg concentrations were significantly higher in coastal regions (28.1 ± 2.9 ng g^−1^, N = 55, with one outlier removed) compared to inland regions (15.5 ± 1.5 ng g^−1^, N = 41) (Fig. 2A, Table S2). Samples of fur and fur-normalized whiskers from adult pumas also had significantly higher mean THg concentrations in coastal areas (1544 ± 151 ng g^−1^, N = 94) compared to inland regions (492 ± 119 ng g^−1^, N = 18) (Fig. 2B, Table S3).

**Figure 3:**
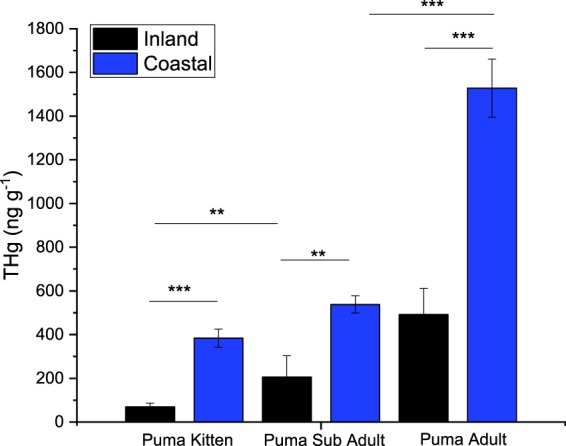
Mean (±1 SE) concentrations of THg in puma samples from California, USA, from three age classes and two geographical regions. Asterisks indicate the p-value from a one-way ANOVA test on the log-transformed concentrations, shown by the extent of the horizontal lines *p = 0.01–0.05, **p = 0.001–0.01, ***p = < 0.001. Sampling for puma was done in 2006–2014.

Different age-classes of pumas were sampled within both coastal and inland regions, and THg concentrations increased with age in both sampling areas (adult > sub-adult > kitten) (Fig. 3). Differences between adult and sub-adult were significant in the coastal region, and differences between kitten and sub-adult were significant in the inland region. Six mother-kitten pairs were also analyzed for THg from the coastal region. Kitten THg concentrations were on average 44.7% ± 9.2% of their mothers. We did not observe any significant differences in THg concentrations between sexes in coastal or inland regions. In all age classes, pumas from the coastal region had significantly higher THg concentrations compared to inland pumas and coastal kittens, on average, were, notably, 5.6 times higher than inland kittens (Fig. 3).

Archived California puma fur samples (1916–1933, Museum of Vertebrate Zoology, University of California, Berkeley, CA, USA) had a mean (±1 SE) THg concentration of 290 ± 42 ng g^−1^ (Fig. 2B). This value was calculated based on the measured MMHg concentration in the archived puma fur, multiplied by the factor 1.15, which was the mean THg/MMHg ratio in the nine modern-day samples analyzed for both Hg species. THg concentrations in archived puma fur were also measured, however, these values were not used due to the high probability of inorganic Hg contamination (mean concentration = 5200 ng g^−1^) which can be common in museum archived vertebrate samples^45^. The mean calculated THg concentration (from MMHg) in archived puma samples was not significantly different compared to the mean from modern-day inland samples, representing a relatively stable background THg concentration over time.

### Methylmercury toxicity and risk to coastal california puma

**Figure 4:**
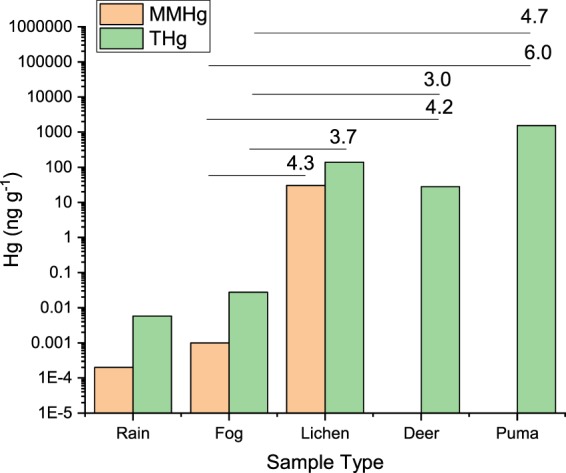
Concentrations of THg and MMHg in rain (sampled 2014–2015), fog (sampled 2014–2015), lichen (sampled 2017), deer (sampled 2008–2012), and puma (sampled 2006–2014). All samples are restricted to locations in the coastal central California, USA region. Fog and rain data are from Weiss-Penzias *et al*.^16^. Bioconcentration factors (defined in the text) are shown for the Hg species and trophic relationship given by the horizontal line.

Bioconcentration factors (BCF) were calculated using methods described by Azad *et al*.^46^, and utilizing the following formula: log ([Hg_organism_]/[Hg_fog_]), where Hg was THg or MMHg (Fig. 4). BCF values indicate how much THg and MMHg can be potentially transferred to lichen, deer, and puma from fog water. The highest BCF found was between puma THg and fog MMHg (6.0). Several authors report that MMHg undergoes more efficient trophic transfer, across a wide array of species, and is more bioavailable compared with THg^46–48^, which is consistent with our hypothesis that fog water MMHg is disproportionately transferred between trophic levels compared with inorganic Hg from other sources.

In the Florida Everglades elevated levels of Hg were detected in puma, however the diet of the Florida pumas included aquatic and fish-eating prey which increased the Hg exposure regime^49,50^. The coastal California pumas in our study, conversely, eat a less diverse diet largely of terrestrial origin. In a previous investigation, we intensively recorded coastal California puma movements and diet, and reported that black-tailed or mule deer made up greater than 90% of the edible biomass for pumas in the Santa Cruz Mountains, and our data showed that coastal pumas were not eating marine derived prey^51^. Studies from other parts of California have also shown deer to be their primary source of prey^52,53^. Since deer fur from coastal California also displayed higher THg concentrations compared to their inland counterparts, and the fact that deer are herbivores foraging on plants and lichen suggests a potential link between fog wet deposition of Hg and the accumulation of Hg in coastal, terrestrial food webs.

While mercury toxicity studies have yet to be conducted in pumas, studies on other carnivores (mink and otter) revealed that brain THg concentrations >3 μg g^−1^ can cause clinical Hg intoxication or subtler neurological impairments that could detrimentally affect survival^54,55^. Assuming that otter and mink brain concentrations of THg are 14% of those in their fur, using the predictions of Eccles *et al*.^56^, concentrations of >21 μg g^−1^ in fur would be considered toxic. Among pumas sampled from coastal regions in this investigation, one individual had a fur sample of >21 μg g^−1^, a puma found dead with no apparent cause of death known at the time.

Sub-lethal effects of Hg are known to occur at lower concentrations than those causing death, and may negatively impact predator population performance. Previous puma investigations have shown that at blood Hg concentrations >250 ng g^−1^, the number of offspring surviving to adulthood per female falls below 1.0, indicating significant, sub-lethal effects and risk to long-term population survival and viability^50,57^. Fur THg concentration in this work, converted to blood THg concentrations using a relationship for Florida panthers^50^, results in a mean calculated blood THg concentration of 73.9 ± 10.3 ng g^−1^, a factor of 3.4 lower than the threshold. However, our samples included two female pumas that had calculated blood THg concentrations of 356 and 276 ng g^−1^, which exceeds the threshold. Further research is needed to better understand the effects of Hg on puma reproduction and how it might interact with other factors such as low genetic diversity, fragmentation and exposure to rodenticides.

### Sources of mercury to coastal food webs

**Figure 5:**
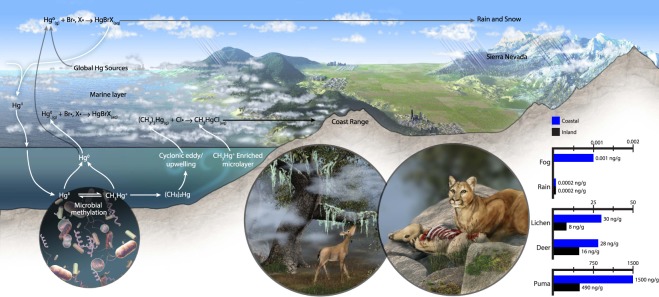
Conceptual diagram showing the hypothesized sources and mechanisms of transfer of organic Hg species from the ocean to the coastal terrestrial food web in central California, USA. Bar charts indicate the mean concentrations of MMHg observed in this work plus fog and rainwater concentration from^16^.

Here we consider the sources of Hg in all its forms potentially contributing to the bioaccumulation of Hg in the coastal vs. inland terrestrial food webs in our study area. We hypothesize that the main source of MMHg in the coastal system is marine fog that carries emissions of organic Hg species from the ocean, as conceptually illustrated in Fig. 5. Note how the coastal range forms a barrier to fog penetration inland, and as the fog is intercepted, wet deposition of marine-derived MMHg occurs. However, our observation of higher MMHg in ocean- vs. bay-facing lichen may have alternate interpretations that should be considered. For example, we do not know the influence of the overall wetness of each habitat, and how this could affect the uptake rates of all forms of Hg to lichen. When the relative humidity is higher, lichen may be more actively taking up Hg. Humidity differences may also affect the rate at which MMHg can be produced endogenously within the lichen due to the presence of symbiotic cyanobacteria that can potentially chemically alter Hg^II^ ^58^. As has been pointed out by previous studies^59^, further research is needed to look for the hgcAB genes within the lichen biome to determine whether *in situ* methylation of Hg can occur.

Our observation that THg concentrations were more elevated in bay-facing lichen samples, whereas the opposite trend was observed for MMHg concentrations, can also have multiple interpretations. One of these is that there is an additional atmospheric deposition source of inorganic Hg being taken up by lichen at the bay-facing sites. We suggest that gaseous oxidized inorganic Hg from the free troposphere may undergo dry deposition to lichen and cause greater accumulation of inorganic Hg at the bay-facing sites. Wright *et al*.^60^ found that Hg dry deposition was a factor of 10 times higher at Lick Observatory in the Diablo Range (Blue Oak Ranch in Fig. 1A), likely due to the prevalence of high pressure subsidence atmospheric conditions^61^, compared with Hg dry deposition at Elkhorn Slough, a site on the Pacific coast within 30 km of our ocean-facing lichen sampling sites. This suggests that the inorganic Hg source from the free troposphere is important for bay-facing sites, whereas ocean-facing sites may be more influenced by Hg° emissions from the ocean and have lower inorganic Hg burdens as a result. We must also consider the potential for the transformation of inorganic to organic Hg within lichen, which may result in higher MMHg but not higher THg, if MMHg in lichen is more volatile and can be lost to the atmosphere over time as Hg°. More laboratory and field studies are needed to understand the dynamics of Hg species in lichen^59^.

We must also consider the potential for dry deposition of gaseous and particulate forms of MMHg, of which there have been no published reports. Throughfall fog water, which drips off of tree foliage, may become enhanced in MMHg due to the washing off of dry deposited MMHg that potentially accumulates during the long rainless season in California, similar to that observed in a mountain-top cloud water study of THg^62^. Additionally, marine fog water contains 90% of its MMHg in the particulate phase^16^ suggesting that either gaseous methylated Hg compounds are taken up by fog droplets and quickly partitioned to particles within the droplet, or MMHg in aerosols are scavenged by the droplet. Because there are no MMHg measurements in aerosols in coastal areas, we cannot determine whether wet or dry deposition of MMHg is a more source to coastal terrestrial ecosystems.

Geogenic sources of Hg from legacy mining activities in the Santa Cruz Mountains must also be considered as a potential source of Hg to the food web of this area^63^, as this impact has been observed near the Idria mine in Slovenia^64^. Results from our lichen samples from the Almaden Quicksilver mining site suggest that mining sources are a large contributor to inorganic Hg, but not MMHg in lichen. However, neither deer fur nor puma fur and whisker THg concentrations were abnormally high in the immediate vicinity of the Almaden Quicksilver mining site (Figs. 1B,C), suggesting that mining Hg may not be particularly mobile in the terrestrial food web compared to atmospheric Hg. The spatial pattern of enhanced puma THg concentrations spanned the entire central California coastal mountain area, with many of these locations are >100 km from Hg mining sites, suggesting that mining sources are of limited importance to the bioaccumulation of Hg in puma in this region.

Lastly, food web variation between coastal and inland puma populations may also potentially explain the differences in concentrations of MMHg between the two geographical areas. However, this is not likely since the diet of pumas in this region is well documented to be fairly simplified especially compared to other puma populations (i.e. limited use of aquatic prey). Moreover, we observed higher MMHg concentrations in coastal areas, compared to inland sites, across all measured trophic levels and this provides multiple lines of evidence that the marine fog input, to the base of the food web, is an important driver of the overall THg bioaccumulation regime in these terrestrial food webs in central coastal California.

## Methods

### Sample origins

Puma whisker and fur samples from the Santa Cruz Mountains and other coastal areas were obtained from our previous research^44,51^, which was archived in a climate-controlled room and available for subsampling fur and whisker for this research (Table S4). The puma capturing, handling and monitoring was done in accordance with protocols approved by the Animal Care and Use Committee at the University of California, Santa Cruz. Approval for capturing, handling and taking samples from pumas was granted by the California Department of Fish and Game. Puma whisker samples from other parts of California were also obtained from necropsies performed at the California Department of Fish and Wildlife (CDFW), who gave approval to this research to take samples for this research. Puma fur was also obtained from pelts in climate-controlled storage at the University of California, Berkeley Museum of Vertebrate Zoology (MVZ), who granted us access to these samples for this research. Mule deer fur samples were obtained from puma kill sites in the Santa Cruz Mountains^44,51^ and from CDFW-led live-captures elsewhere. All fur and whisker samples were put into paper coin envelopes or polyethylene bags. Lichen samples were collected from natural reserves managed by the University of California, the State of California, the City of Santa Cruz, and the County of Santa Clara. Lichens were collected using trace-metal clean sampling techniques, including clean hands-dirty hands, wiping cutting tools with methanol between samples, and sample storage in two polyethylene storage bags.

### Study area

Puma and mule deer samples were collected within selected regions of California (Fig. 2). A distinction was made between samples that came from marine fog-influenced areas (“coastal”) and non-marine foggy inland areas (“inland”). Marine fog influenced coastal areas were determined to have greater than >5 hrs/day average summertime fog and low-cloud cover based on maps published in a previous work^19^. These regions have summertime weather conditions with negligible rainfall, yet a near-daily influence of marine stratus clouds, especially at night. Marine stratus cloud tops in this region typically have heights of 400–600 m, corresponding to cloud thicknesses of 200–400 m^21,65^. During the months between May and September, coastal locations and ocean-facing ridges are particularly impacted by marine stratus clouds with midday overcast occurring on about 50% of the days^21^. The elevation band between 400 and 500 m experiences the greatest amount of summertime wet deposition^22^. The east side of the range which faces the southern San Francisco Bay receives significantly less marine layer influence and fog drip, even though the wintertime precipitation rates on both sides of the range are similar at locations with similar elevation^21^. Fog water deposition can vary 2–3 orders of magnitude as a result of microclimates, changes in exposure and vegetation type^21,22^. Thus, within the coastal region, a further distinction was made between the ocean-facing and bay- facing sample collection sites, using the summit of the Santa Cruz Mountains as a boundary between these sub-regions.

The puma’s diet across California consists primarily of black-tailed or mule deer (*Odocoileus hemionus*)^51^. Pumas that live near the ocean in the Santa Cruz Mountains have been the subject of intense study of movement and diet^51^. From this, it was observed that pumas did not travel to the ocean and there was no marine-derived prey in their diet.

Lace lichen (*Ramalina menziesii*) is a symbiosis between a green alga and a fungus and is widely distributed across the state of California, growing on the understory of trees and bushes. Lace lichen has been utilized to effectively track and chronicle a century of atmospheric lead contamination in the Santa Cruz Mountains^40^.

### Analysis of total mercury and methylmercury

THg was quantified using a direct mercury analyzer (DMA) (Milestone Corp.) following EPA method 7473^66^. Puma fur and whiskers, and deer fur were not rinsed nor freeze dried prior to analyses. We estimated that rinsing was an unnecessary step for this analysis since the puma and deer samples obtained were free of visible particles. As justification for not rinsing, we observed a very tight relationship in THg concentrations paired whisker and fur samples from several individual pumas (Fig. S2). If surface contamination of the samples were significant, we would expect to see a much noisier relationship among these data. Lichen samples were homogenized with liquid nitrogen and an acid-washed mortar and pestle, freeze dried in a lyophilizer in 20 mL glass scintillation vials, then weighed into a nickel boat and analyzed. The accuracy of the DMA was evaluated by quantifying the THg concentration of various certified reference materials (CRMs) between runs of <20 unknown samples. Peach leaves (NIST 1547), apple leaves (NIST 1515), dogfish muscle (DORM-4), plankton (BCR-414) and sediment (BCR-320-R) CRM’s were used for QA/QC. The mean recovery of THg was 91.6 ± 1.5% (N = 77) and the unknown sample THg concentrations were not corrected for this bias. Duplicate CRM samples had THg σ/mean values of 11% (N = 15). Instrument nickel-boat blanks were on average 0.02 ng THg, resulting in a method detection limit of 0.02 ng g^−1^. The instrument detection limit is 0.005 ng Hg.

Monomethyl Hg (MMHg) was quantified using a Tekran© 2500 detector and EPA method 1630^67^, which involves treating a sample solution at pH 5 with sodium tetraethylborate (NaTEB), purging the resulting ethylmethyl Hg onto a solid adsorbent trap filled with Tenax and thermally desorbing onto a GC followed by pyrolysis and detection with by cold vapor atomic fluorescence (CVAFS). Sample preparation for MMHg analysis of the lichen samples involved digesting about 0.5 g of the homogenized and freeze-dried material in 2 mL of 25% KOH/methanol at 60 °C for 4 hours^68^. The resultant mixture was then diluted to 10 mL with 18.2 MΩ deionized water (MilliQ), instead of all methanol, as specified in^68^ due to the small amounts of lichen needed (~0.2 g) for analysis. Next 100 μL of this digestate was added to ~100 mL of MilliQ water, adjusted to pH 5 with acetate buffer and treated with NaTEB in accordance with EPA method 1630. The accuracy of this method was assessed by determining the concentration of MMHg in CRM (DORM-4) with a certified MMHg concentration of 355 ng g^−1^. This comparison yielded an overall accuracy of 90.2 ± 6.3% (N = 6). The percent recovery of MMHg in the DORM-4 CRM sample was checked using both the KOH digestion protocol and another commonly used method: digesting in 5 mL of 4.5 M HNO_3_ in a water bath at 60 °C for 12 hours^69^. Previous studies have suggested that the KOH method can have poor recoveries in certain matrices due to interference from organic compounds^70,71^. However, no significant difference was observed in the % recovery of MMHg in DORM-4 samples treated with either KOH or HNO_3_. A small number of puma whisker and fur (N = 9) and deer fur (N = 4) samples were then digested using the HNO_3_ digestion protocol. The instrument detection limit was 0.14 ng Hg and the method detection limit, defined as three times the standard deviation of the digestion blank was 0.29 ng g^−1^. The average value of the digestion blanks was 0.51 ng g^−1^ (N = 7) and the duplicate CRM analyses had a σ/mean value of 14% (N = 6).

Speciation of Hg was evaluated in nine individual adult pumas (two whisker and seven fur samples) sampled from across the coastal and inland regions and %MMHg/THg was 87.2% ± 12.2% demonstrating that MMHg, the highly neurotoxic form, was the dominant species of mercury in the whisker and fur samples. It also demonstrates that using THg as a convenient measure of MMHg in pumas is reasonable.

### Statistical analyses

The individual organism, lichen, deer or puma, was the biological unit of replication. These data were grouped according to geographic sub-region for lichen, deer, and puma, and by age class and sex for puma (Table 1). The raw concentration data were examined for normality using a Shapiro-Wilks test (OriginLab software (OriginLab Corp., Northampton, MA USA)) and in most cases, the distributions were skewed. Thus, a subsequent one-sample ANOVA analysis (OriginLab) to determine significant difference of the means between two subsets of concentration data, was carried out using log-transformed distributions of concentrations. Each subset of data was also analyzed for outlier concentrations using a Grubb’s test^72^ (OriginLab) and these data are identified by an asterisk in Table 1. The outliers were removed before calculating the means, medians, standard deviations, and standard errors. Statistical significance for the ANOVA test was accepted at *P* < 0.05^73^, and for the Grubb’s outlier test significance was accepted at *P* < 0.01. The residuals were analyzed (OriginLab) for constant variances, if there was drift, or if the error term was independent. In all cases, no pattern in the residuals was detected. The Moran’s I test for spatial autocorrelation (SA) was performed^74^ using R v. 3.6.1 (R foundation for Statistical Computing) and the null hypothesis of the existence of significant autocorrelation was rejected when p > 0.05. According to this criteria, SA could be rejected for all data sets except for MMHg concentrations in lichen. This finding increased the chance of a Type I error (incorrect rejection of a null hypothesis) and where this occurred, we must interpret the results with caution. However, SA in the lichen MMHg concentrations is also a natural outcome of the study design due to the ocean-facing sites being more clustered in one region compared to the bay-facing sites, which were more spread out. In a previous study, a random selection of data at a 1 km spatial resolution was carried out to minimize SA^75^, but due to the small number of lichen sampling sites in this study, this was not possible.

## Supplementary information

Supporting Information
