# Supplementary material for: Marine fog inputs appear to increase methylmercury bioaccumulation in a coastal terrestrial food web
Source: Sci Rep. 2019 Nov 26;9:17611. doi: 10.1038/s41598-019-54056-7 (PMC6879473; doi:10.1038/s41598-019-54056-7)
Supplement: Supplementary file 1 — Supporting Information [file 41598_2019_54056_MOESM1_ESM.docx]

**Supporting Information for Weiss-Penzias et al. “Marine fog inputs appear to increase methylmercury bioaccumulation in a coastal terrestrial food web”**

Peter S. Weiss-Penzias^1^, Michael S. Bank^2,3^, Deana L. Clifford^4,5^, Alicia Torregrosa^6^, Belle Zheng^1^, Wendy Lin^1^ and Christopher C. Wilmers^7^

^1^ Department of Microbiology and Environmental Toxicology, University of California - Santa Cruz, CA USA

^2^ Institute of Marine Research, Department of Contaminants and Biohazards, Bergen, Norway

^3^ University of Massachusetts, Department of Environmental Conservation, Amherst, MA USA

^4^ Wildlife Investigations Lab, California Department of Fish and Wildlife, Rancho Cordova, CA USA

^5^ University of California, School of Veterinary Medicine, Department of Medicine and Epidemiology, Davis, CA USA

^6^ United States Geological Survey, Western Geographic Science Center, Menlo Park, CA USA

^7^ Environmental Studies Department, University of California - Santa Cruz, CA USA

**Figure Legends**

Figure S-1: Monomethylmercury (MMHg) log-transformed concentrations in lace lichen (*Ramalina menziesii*) samples from locations in Table 2 (California, USA), plotted versus (A) distance to the ocean and (B) mean summertime fog and low cloud coverage frequency (FLCC) from (19). Linear fit statistics on the log transformed data are shown. Samples were collected during the summer-fall of 2017.

Figure S-2: Total Hg concentrations in fur and whisker samples from 25 individual pumas sampled during 2006-2012 and from locations in California, USA. The linear relationship is: THg_fur_ = 1.83 x THg_whiskers_ (R^2^ = 0.97, *P* < 0.0001). This slope was then used to convert whisker THg concentrations to their fur equivalents.

**Table Headings**

Table S-1: Total Hg and monomethyl Hg concentrations in lichen (*Ramalina menziesii*) samples separated by sub-region in California, USA, including information on sampling site distance to the ocean, and the mean summertime fog and low coastal cloud frequency (FLCC) (19). An asterisk indicates the value is an outlier according to a Grubb’s test.

Table S-2: Total Hg concentrations in deer fur samples from California, USA, sampled during 2008-2012.

Table S-3: Total Hg concentrations in puma fur and whisker-normalized-to-fur samples from California, USA, sampled during 2006-2012.

Table S-4: Summary of biotic samples taken from both coastal and inland regions of California, USA.

Figure S-1: Monomethylmercury (MMHg) log-transformed concentrations in lace lichen (*Ramalina menziesii*) samples from locations in Table 2 (California, USA), plotted versus (A) distance to the ocean and (B) mean summertime fog and low cloud coverage frequency (FLCC) from (19). Linear fit statistics on the log transformed data are shown. Samples were collected during the summer-fall of 2017.


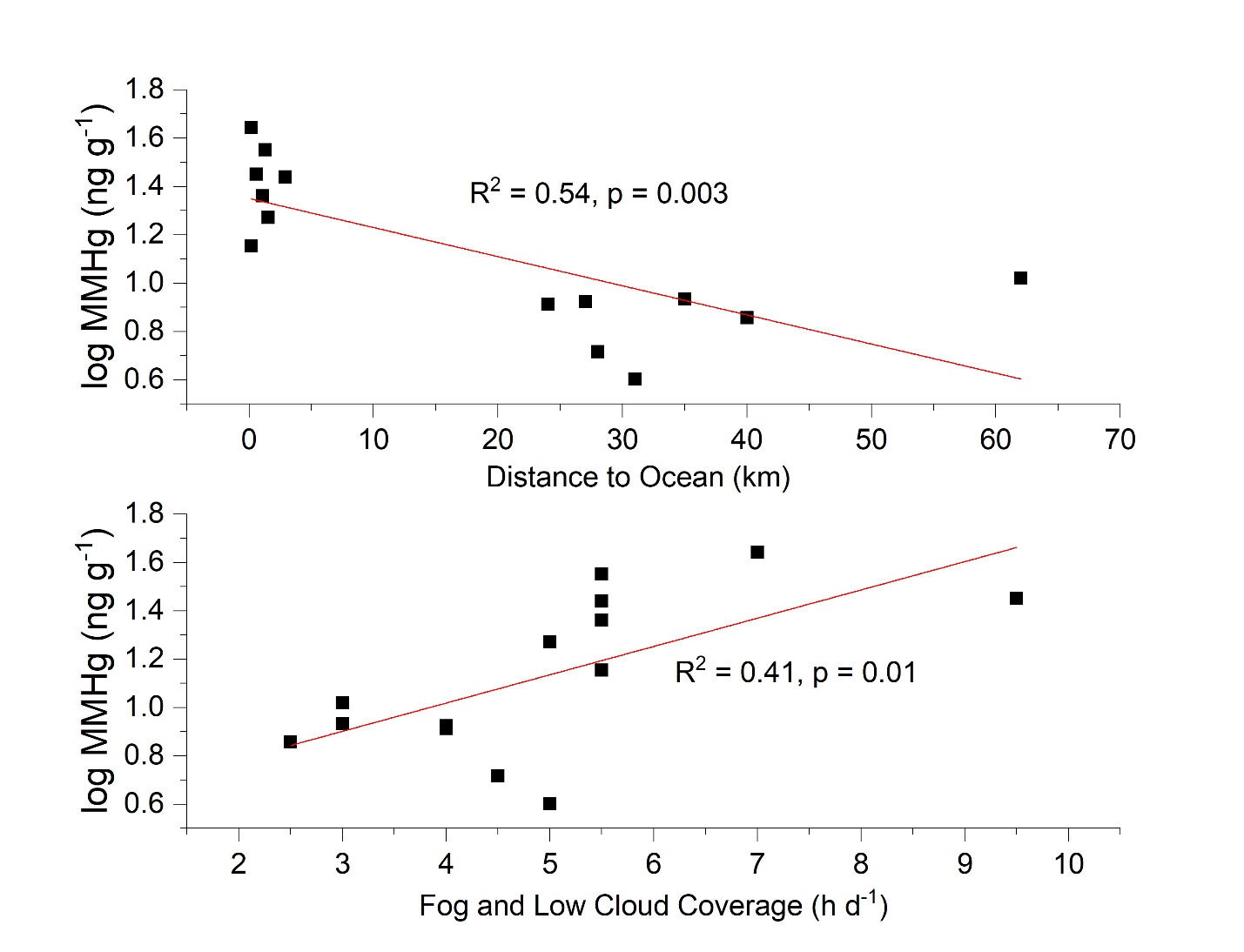


Figure S-2: Total Hg concentrations in fur and whisker samples from 25 individual pumas sampled during 2006-2012 and from locations in California, USA. The linear relationship is: THg_fur_ = 1.83 x THg_whiskers_ (R^2^ = 0.97, *P* < 0.0001). This slope was then used to convert whisker THg concentrations to their fur equivalents.


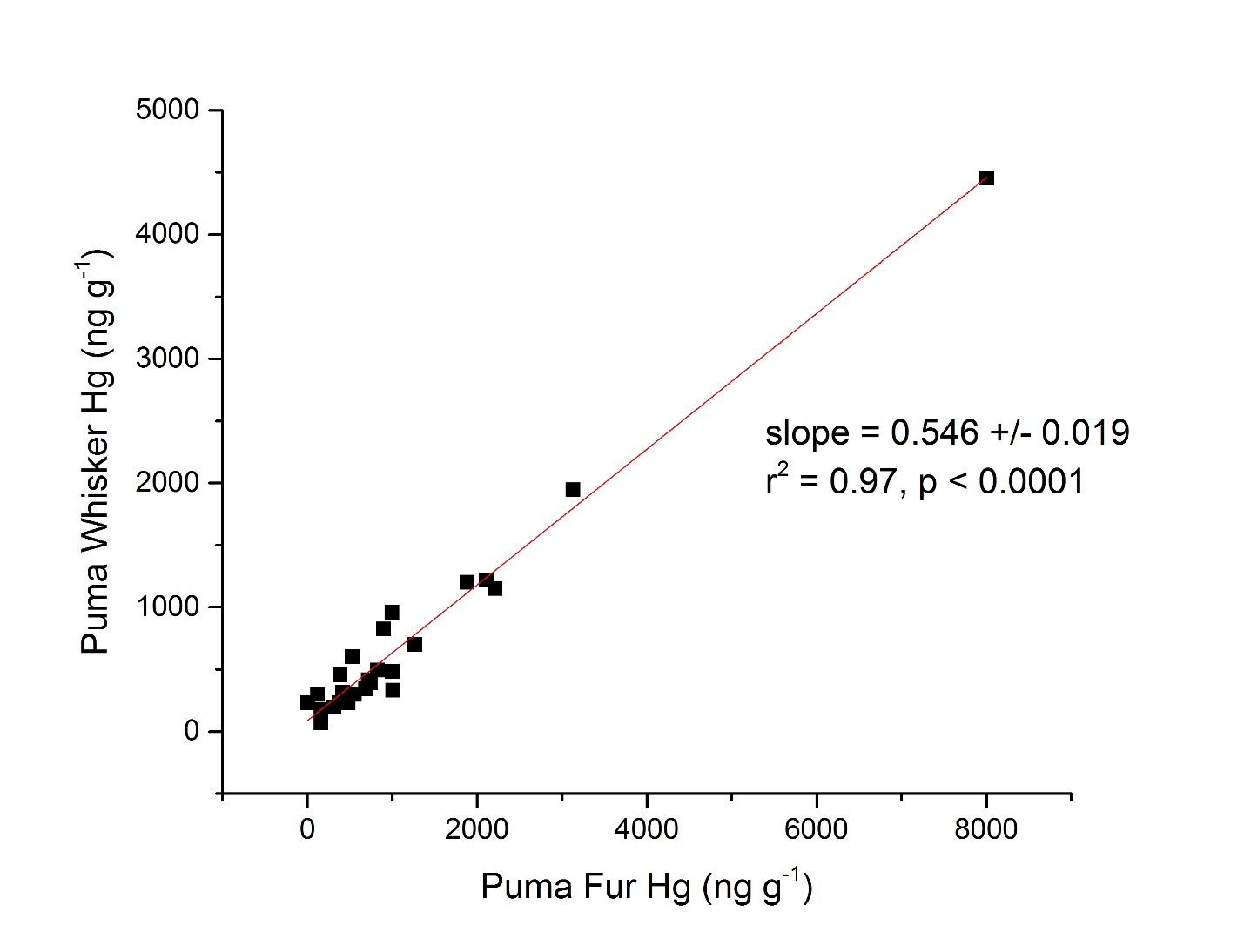


Table S-1: Total Hg and monomethyl Hg concentrations in lichen (*Ramalina menziesii*) samples separated by sub-region in California, USA, including information on sampling site distance to the ocean, and the mean summertime fog and low coastal cloud frequency (FLCC) (19). An asterisk indicates the value is an outlier according to a Grubb’s test. These samples were collected during the summer-fall of 2017.

| Region | Location | Dist. To Coast (km) | FLCC | THg (ng g^-1^) | | MMHg (ng g^-1^) | | MMHg/ THg  (%) |
| --- | --- | --- | --- | --- | --- | --- | --- | --- |
|  |  |  | h d^-1^ | mean | SE | mean | SE |  |
| Ocean-facing | Younger Lagoon | 0.2 | 5.5 | 49.5 | - | 14.3 | 9.7 | 28.8 |
|  | Greyhound Rock | 0.2 | 7.0 | 158.6 | 5.9 | 44.0 | 12.0 | 27.6 |
|  | Ano Nuevo | 0.6 | 9.5 | 144.6 | 9.0 | 28.2 | 6.2 | 19.3 |
|  | Wilder Ranch | 1.1 | 5.5 | 143.8 | 33.1 | 23.0 | 5.7 | 17.7 |
|  | Davenport | 1.3 | 5.5 | 105.3 | - | 35.6 | 5.4 | 33.8 |
|  | Nisene Marks | 1.5 | 5.0 | 140.2 | 12.2 | 18.7 | 13.0 | 13.3 |
|  | Moore Creek | 2.9 | 5.5 | 132.7 | 11.8 | 27.5 | 6.7 | 20.8 |
|  | Mean | 1.1 | 6.2 | 138.1 | 11.5 | 30.3 | 3.5 | 23.0 |
| Bay-facing | Alamaden Quicksilver | 24 | 4.0 | 1097* | 58.2 | 8.2 | 3.3 | 0.8 |
|  | Calero Res | 27 | 4.0 | 160.9 | 2.1 | 8.4 | 2.9 | 5.2 |
|  | Lexington Res | 28 | 4.5 | 150.1 | 4.7 | 5.2 | 1.9 | 4.0 |
|  | Stevenson's Creek | 31 | 5.0 | 85.7 | 4.0 | 4.0 | 2.4 | 4.6 |
|  | Anderson Lake | 35 | 3.0 | 154.8 | 1.2 | 8.6 | 3.3 | 5.5 |
|  | Henry Coe | 40 | 2.5 | 280.8 | 5.3 | 7.2 | 2.3 | 2.5 |
|  | Blue Oak Ranch | 62 | 3.0 | 242.5 | 20.9 | 10.5 | 2.2 | 4.5 |
|  | Mean | 35.3 | 3.7 | 333.6 | 12.2 | 8.2 | 0.8 | 3.9 |
|  | Mean w/o Almaden |  |  | 206.3 | 20.6 |  |  | 4.4 |

Table S-2: Total Hg concentrations in deer fur samples from California, USA, sampled during 2008-2012.

| Animal ID | Latitude | Longitude | Region | Sub-Region | THg (ng/g) |
| --- | --- | --- | --- | --- | --- |
| DR14105 | 38.764 | -119.961 | inland |  | 20.4 |
| DR13031 | 39.499 | -119.983 | inland |  | 6.7 |
| DR13027 | 39.379 | -120.127 | inland |  | 4.4 |
| DR13025 | 39.37 | -120.071 | inland |  | 2.8 |
| DR13015 | 38.676 | -119.949 | inland |  | 13.3 |
| DR13006 | 39.449 | -120.234 | inland |  | 25.5 |
| DR13004 | 39.465 | -120.29 | inland |  | 18.8 |
| DR13003 | 39.465 | -120.29 | inland |  | 8.3 |
| DR10017 | 39.396 | -120.163 | inland |  | 12.5 |
| DR10015 | 39.355 | -120.121 | inland |  | 20.7 |
| dr13011 | 39.492 | -120.393 | inland |  | 16.7 |
| dr13008 | 39.482 | -120.304 | inland |  | 9 |
| DE13014 | 35.161 | -115.378 | inland |  | 9.5 |
| de13010 | 35.123 | -115.441 | inland |  | 9.8 |
| de13003 | 35.386 | -115.246 | inland |  | 19.2 |
| de12016 | 35.14 | -115.404 | inland |  | 11.5 |
| de12014 | 35.076 | -115.47 | inland |  | 12.8 |
| DE12011 | 35.298 | -115.508 | inland |  | 8.1 |
| de12009 | 35.291 | -115.549 | inland |  | 15.3 |
| de12003 | 35.364 | -115.151 | inland |  | 14.2 |
| de11030 | 35.289 | -115.585 | inland |  | 9.3 |
| DE11006 | 35.289 | -115.585 | inland |  | 10.6 |
| dc11041 | 37.858 | -119.99 | inland |  | 29.7 |
| dc11039 | 37.885 | -119.965 | inland |  | 4.1 |
| DC11011 | 37.9 | -120 | inland |  | 7.8 |
| dc11010 | 37.917 | -119.981 | inland |  | 13.4 |
| dc110003 | 37.895 | -120.033 | inland |  | 4.7 |
| dc10048 | 37.845 | -120.214 | inland |  | 17.8 |
| dc10034 | 37.845 | -120.197 | inland |  | 16 |
| dc10029 | 37.849 | -120.22 | inland |  | 14.1 |
| dc10020 | 37.855 | -120.217 | inland |  | 8.9 |
| db11089 |  |  | inland |  | 26.4 |
| db11054 | 39.573 | -122.896 | inland |  | 31.9 |
| DB11053 | 39.575 | -122.94 | inland |  | 18.7 |
| db11051 | 39.609 | -122.947 | inland |  | 51.8 |
| DB11050 | 39.587 | -122.889 | inland |  | 9.1 |
| DB11008 | 40.573 | -124.27 | inland |  | 28.9 |
| DB10031 | 39.795 | -122.956 | inland |  | 13 |
| DB10020 | 39.718 | -122.901 | inland |  | 30 |
| db10016 | 39.747 | -122.888 | inland |  | 14.4 |
| 10017 | 37.061 | -122.209 | coastal | ocean-facing | 82.5 |
| 10028 | 37.016 | -122.163 | coastal | ocean-facing | 27.7 |
| 50050 | 37.126 | -121.764 | coastal | bay-facing | 15.6 |
| 70172 | 37.174 | -121.93 | coastal | bay-facing | 19.2 |
| 90021 | 36.963 | -121.832 | coastal | ocean-facing | 35.1 |
| 110100 | 37.229 | -122.014 | coastal | bay-facing | 15.3 |
| 110181 | 37.205 | -121.99 | coastal | bay-facing | 49.2 |
| 190115 | 37.22 | -122.007 | coastal | bay-facing | 54.9 |
| 220355 | 37.222 | -122.091 | coastal | bay-facing | 15.2 |
| 230012 | 37.013 | -121.924 | coastal | ocean-facing | 66.3 |
| 230016 | 37.008 | -121.916 | coastal | ocean-facing | 48.9 |
| 230149 | 36.996 | -121.884 | coastal | ocean-facing | 8 |
| 250072 | 36.99 | -122.093 | coastal | ocean-facing | 26.1 |
| 250078 | 36.985 | -122.123 | coastal | ocean-facing | 63.1 |
| 270229 | 37.122 | -121.849 | coastal | bay-facing | 92.9 |
| 280097 | 37.197 | -121.861 | coastal | bay-facing | 57.1 |
| DB11042 | 37.072 | -121.478 | coastal | bay-facing | 21.5 |
| DB11076 | 37.566 | -122.395 | coastal | bay-facing | 11.2 |
| DB11077 | 37.501 | -122.312 | coastal | bay-facing | 13.8 |
| DB11079 | 37.573 | -122.402 | coastal | bay-facing | 9.2 |
| DB11084 | 37.56 | -122.392 | coastal | bay-facing | 10.1 |
| DB12002 | 37.496 | -122.314 | coastal | bay-facing | 30.4 |
| DB12064 | 37.072 | -121.478 | coastal | bay-facing | 30.6 |
| DB13004 | 37.293 | -121.754 | coastal | bay-facing | 12.6 |
| DB13007 | 37.293 | -121.754 | coastal | bay-facing | 16.5 |
| DB13011 | 37.293 | -121.754 | coastal | bay-facing | 32.3 |
| DB13012 | 37.293 | -121.754 | coastal | bay-facing | 11.7 |
| DB13013 | 37.293 | -121.754 | coastal | bay-facing | 6.1 |
| DB13016 | 37.293 | -121.754 | coastal | bay-facing | 10 |
| DB13020 | 37.293 | -121.754 | coastal | bay-facing | 6.9 |
| DB13023 | 37.293 | -121.754 | coastal | bay-facing | 11.1 |
| DB13024 | 37.293 | -121.754 | coastal | bay-facing | 2.8 |
| DB13026 | 37.293 | -121.754 | coastal | bay-facing | 13.8 |
| DB13027 | 37.293 | -121.754 | coastal | bay-facing | 22.1 |
| DB13031* | 37.293 | -121.754 | coastal | bay-facing | 186.3 |
| DB13032 | 37.293 | -121.754 | coastal | bay-facing | 9.8 |
| DB13037 | 37.293 | -121.754 | coastal | bay-facing | 28.8 |
| DB13038 | 37.293 | -121.754 | coastal | bay-facing | 28 |
| DB13040 | 37.293 | -121.754 | coastal | bay-facing | 9.9 |
| dh01 | 37.17 | -121.948 | coastal | bay-facing | 4.7 |
| dh02 | 37.291 | -122.055 | coastal | bay-facing | 11.4 |
| dh03 | 37.205 | -121.99 | coastal | bay-facing | 6.8 |
| dh04 | 37.202 | -122.006 | coastal | bay-facing | 7.9 |
| dh05 | 37.213 | -122.035 | coastal | bay-facing | 14.2 |
| dh06 | 37.229 | -122.105 | coastal | bay-facing | 23 |
| dh07 | 37.001 | -121.911 | coastal | ocean-facing | 15.4 |
| dh08 | 36.993 | -122.115 | coastal | ocean-facing | 18.6 |
| dh09 | 36.987 | -122.106 | coastal | ocean-facing | 67.3 |
| dh10 | 37.193 | -121.857 | coastal | bay-facing | 64.4 |
| dh11 | 37.111 | -122.192 | coastal | ocean-facing | 28.7 |
| dh12 | 36.977 | -122.103 | coastal | ocean-facing | 53.4 |
| dh13 | 37.047 | -122.115 | coastal | ocean-facing | 33 |
| dh14 | 36.996 | -122.047 | coastal | ocean-facing | 28.8 |
| dh15 | 37.071 | -122.243 | coastal | ocean-facing | 58 |
| dh16 | 37.026 | -122.197 | coastal | ocean-facing | 53 |
| dh17 | 37.048 | -122.214 | coastal | ocean-facing | 28.5 |

Table S-3: Total Hg concentrations in puma fur and whisker-normalized-to-fur samples from California, USA, sampled during 2006-2012.

| Animal ID | Latitude | Longitude | Region | Sub-Region | Sample | Sex | Age Class | Fur-Normalized THg ng/g |
| --- | --- | --- | --- | --- | --- | --- | --- | --- |
|  |  |  |  |  | Type |  |  |  |
| 23745 | 37.536 | -119.704 | museum |  | fur | M | adult | 114.8 |
| 31252 | 38.995 | -120.622 | museum |  | fur | M | adult | 370.4 |
| 33471 | 37.757 | -119.959 | museum |  | fur | M | adult | 258.5 |
| 33553 | 38.76 | -122.612 | museum |  | fur | F | adult | 328 |
| 33554 | 38.73 | -122.642 | museum |  | fur | F | adult | 266.4 |
| 35375 | 40.888 | -122.116 | museum |  | fur | F | adult | 158.4 |
| 37423 | 32.733 | -116.576 | museum |  | fur | M | adult | 373.2 |
| 37424 | 32.893 | -116.501 | museum |  | fur | F | adult | 213.6 |
| 37425 | 32.913 | -116.443 | museum |  | fur | M | adult | 524 |
| holm 2-21-2015 | 39.128 | -121.027 | inland |  | whisker | M | adult | 484.5 |
| n14-256 | 38.175 | -120.377 | inland |  | whisker | M | adult | 254.4 |
| n14-257 | 38.361 | -120.671 | inland |  | whisker | M | kitten | 59.2 |
| n14-517 | 38.372 | -120.693 | inland |  | whisker | M | adult | 550.5 |
| n14-518 | 38.395 | -120.469 | inland |  | whisker | M | sub adult | 99.4 |
| n14-519 | 38.21 | -120.533 | inland |  | whisker | F | sub adult | 256.1 |
| n14-520 | 38.3 | -120.604 | inland |  | whisker | F | adult | 122.3 |
| n14-521 | 38.372 | -120.508 | inland |  | whisker | F | adult | 196.5 |
| n14-522 | 38.261 | -120.577 | inland |  | whisker | M | sub adult | 142.4 |
| n14-523 | 38.372 | -120.693 | inland |  | whisker | F | adult | 160 |
| n14-524 | 38.21 | -120.533 | inland |  | whisker | M | adult | 499.3 |
| Z16-1238 | 33.079 | -116.602 | inland |  | whisker | M | adult | 290.6 |
| Z16-1346 | 38.192 | -120.829 | inland |  | whisker | M | adult | 771.9 |
| Z16-409 | 41.643 | -120.217 | inland |  | whisker | F | sub adult | 273.8 |
| Z17-0021 | 37.871 | -120.433 | inland |  | whisker | F | adult | 353.7 |
| Z17-0022 | 37.485 | -119.966 | inland |  | whisker | M | adult | 157.1 |
| Z17-0036 | 39.101 | -120.953 | inland |  | whisker | M | kitten | 59.4 |
| Z17-0155 | 39.676 | -120.241 | inland |  | whisker | M | adult | 646.1 |
| Z17-0162 | 32.853 | -116.616 | inland |  | whisker | F | sub adult | 124.1 |
| Z17-0168 | 38.196 | -120.681 | inland |  | whisker | F | adult | 1946.3 |
| Z17-0197 | 38.761 | -120.587 | inland |  | whisker | F | adult | 207 |
| Z17-0209 | 38.73 | -120.799 | inland |  | whisker | F | kitten | 26.8 |
| Z17-0317 | 37.419 | -118.588 | inland |  | whisker | M | adult | 72 |
| Z17-0426 | 41.608 | -122.84 | inland |  | whisker | M | adult | 1574.4 |
| Z17-0451 | 38.907 | -120.839 | inland |  | whisker | F | kitten | 68.3 |
| Z17-0466 | 38.04 | -120.4 | inland |  | whisker | M | sub adult | 337.7 |
| Z17-0467 | 37.983 | -120.382 | inland |  | whisker | F | adult | 246.6 |
| Z17-0468 | 37.903 | -120.302 | inland |  | whisker | F | kitten | 132.9 |
| Z17-1316 |  |  | inland |  | whisker | M | adult | 319.2 |
| 10F_5May15 | 37.076 | -122.172 | coastal | ocean-facing | fur | F | adult | 914.3 |
| 11F_1Dec11 | 37.171 | -121.897 | coastal | bay-facing | fur | F | adult | 411.1 |
| 13F_11Jan10 | 37.213 | -121.934 | coastal | bay-facing | fur | F | adult | 850.2 |
| 14F_18Mar2010 |  |  | coastal |  | fur |  | kitten | 160.1 |
| 15F_28Apr10 | 37.137 | -122.067 | coastal |  | fur | F | sub adult | 306.1 |
| 16M_7May10 | 37.137 | -122.067 | coastal | ocean-facing | fur | M | adult | 683.2 |
| 18F_12Nov10 | 37.073 | -121.925 | coastal | ocean-facing | fur | F | adult | 552.7 |
| 19F_23Aug2012 | 37.078 | -122.111 | coastal |  | whisker |  | kitten | 581.4 |
| 20F_13Dec10 | 36.976 | -121.792 | coastal | ocean-facing | fur | M | adult | 965.5 |
| 21M_22Jan11 | 37.228 | -122.172 | coastal | ocean-facing | fur | M | adult | 269.4 |
| 22M_20Apr11 |  |  | coastal |  | fur | M | adult | 1356.4 |
| 23 F kitten 2014 |  |  | coastal |  | fur |  | kitten | 375.7 |
| 23F kitten 2012 |  |  | coastal |  | fur |  | kitten | 326.9 |
| 23F_28Mar2012 | 37.01 | -121.938 | coastal | ocean-facing | fur | F | adult | 645.2 |
| 23F_9May11 | 37.088 | -122.044 | coastal | ocean-facing | fur | F | adult | 553.6 |
| 24F_17May11 | 37.13 | -121.777 | coastal | bay-facing | fur | F | adult | 900.1 |
| 24F_26Aug2011 |  |  | coastal |  | fur |  | kitten | 577.8 |
| 24F_26Aug2011 |  |  | coastal |  | fur |  | kitten | 416.7 |
| 25F_22Sep2011 |  |  | coastal |  | fur |  | kitten | 378.4 |
| 25F_22Sep2012 |  |  | coastal |  | fur |  | kitten | 471.1 |
| 25F_3Aug11 | 36.987 | -122.12 | coastal |  | fur | F | adult | 1309.7 |
| 25F_3Feb15 | 36.961 | -122.091 | coastal |  | fur | F | adult | 6530.2 |
| 26M_11Nov14 | 37.195 | -121.909 | coastal | bay-facing | fur | M | adult | 746.9 |
| 28F 2014* |  |  | coastal |  | fur |  | kitten | 4495.4 |
| 28F_26Nov13 | 37.202 | -121.905 | coastal | bay-facing | fur | F | adult | 620 |
| 29F_10Dec2013 |  |  | coastal |  | fur |  | kitten | 540.6 |
| 29F_19Mar13 | 37.04 | -122.128 | coastal | ocean-facing | fur | F | adult | 922.7 |
| 29F_4Sep14 | 37.04 | -122.118 | coastal | ocean-facing | fur | F | adult | 805.4 |
| 34M_25Nov12 | 37.021 | -121.81 | coastal | ocean-facing | fur | M | adult | 1635.8 |
| 35M_6Apr12 | 37.108 | -122.015 | coastal |  | fur | M | sub adult | 141.1 |
| 44M_10Dec2013 |  |  | coastal |  | fur |  | kitten | 75.8 |
| 45M kitten |  |  | coastal |  | fur |  | kitten | 118.9 |
| 52F_17Jan16 | 37.125 | -122.2 | coastal | ocean-facing | fur | F | adult | 1064.7 |
| 53F_14Sep15 | 37.094 | -121.891 | coastal | ocean-facing | fur | F | adult | 633.6 |
| 54M 4-8-15 | 37.099 | -121.969 | coastal |  | fur | M | sub adult | 839.8 |
| 55F 4-14-15 | 37.233 | -122.2 | coastal | ocean-facing | fur | F | adult | 2810.9 |
| 56M 5-18-15 | 37.575 | -122.423 | coastal |  | fur | M | sub adult | 615.4 |
| 57M kitten |  |  | coastal |  | fur |  | kitten | 194.7 |
| 62M 10-20-15 | 37.016 | -122.155 | coastal | ocean-facing | fur | M | adult | 1260.2 |
| 63M_22Oct2015 |  |  | coastal |  | fur |  | kitten | 483.4 |
| 64M_22Oct2015 |  |  | coastal |  | fur |  | kitten | 546 |
| 65F_22Oct2015 |  |  | coastal |  | fur |  | kitten | 517.1 |
| 66M 10-27-15 | 37.171 | -121.896 | coastal | bay-facing | fur | M | adult | 1065.5 |
| 68M 12-9-15 | 37.205 | -121.917 | coastal |  | fur | M | sub adult | 280.4 |
| 7F_30Oct2010 | 37.195 | -121.946 | coastal | bay-facing | fur | F | adult | 325.7 |
| 9M_24Jan11 | 37.132 | -122.168 | coastal |  | fur | M | sub adult | 336.4 |
| ML-02 | 36.973 | -121.721 | coastal | ocean-facing | whisker |  | adult | 1555.5 |
| ML-03 |  |  | coastal |  | whisker |  | adult | 603.9 |
| ML-04 | 37.023 | -121.736 | coastal | ocean-facing | whisker |  | adult | 1335.9 |
| ML-05 | 37.222 | -121.862 | coastal | bay-facing | whisker |  | adult | 6405 |
| ML-06 | 35.389 | -120.609 | coastal |  | whisker |  | adult | 6020.7 |
| ML-07* | 36.965 | -121.767 | coastal |  | whisker |  | adult | 22051.5 |
| ML-08 | 37.593 | -121.889 | coastal | bay-facing | whisker |  | adult | 988.2 |
| ML-09 | 35.487 | -120.671 | coastal |  | whisker |  | adult | 1226.1 |
| ML-10 | 37.694 | -122.086 | coastal | bay-facing | whisker |  | adult | 677.1 |
| ML-11 | 37.083 | -122.051 | coastal | ocean-facing | whisker |  | adult | 366 |
| ML-12 | 35.97 | -121.451 | coastal |  | whisker |  | adult | 2818.2 |
| ML-13 | 37.311 | -121.893 | coastal | bay-facing | whisker |  | adult | 2635.2 |
| ML-14 | 36.675 | -121.655 | coastal |  | whisker |  | adult | 1207.8 |
| ML-15 | 37.181 | -121.994 | coastal | bay-facing | whisker |  | adult | 732 |
| ML-16 |  |  | coastal |  | whisker |  | adult | 2250.9 |
| ML-17 | 37.003 | -121.569 | coastal | bay-facing | whisker |  | adult | 1555.5 |
| ML-18 | 37.169 | -121.883 | coastal | bay-facing | whisker | M | adult | 2287.5 |
| ML-19 | 37.169 | -121.883 | coastal | bay-facing | whisker | M | adult | 2324.1 |
| ML-20 | 37.203 | -121.914 | coastal | bay-facing | whisker | M | adult | 5398.5 |
| ML-21 | 37.203 | -121.914 | coastal | bay-facing | whisker | M | adult | 1793.4 |
| ML-22 | 37.121 | -122.204 | coastal | ocean-facing | whisker | M | adult | 4556.7 |
| ML-23 | 37.121 | -122.204 | coastal | ocean-facing | whisker | M | adult | 951.6 |
| ML-24 | 37.121 | -122.204 | coastal | ocean-facing | whisker | F | adult | 1701.9 |
| ML-25 | 37.121 | -122.204 | coastal | ocean-facing | whisker | F | adult | 1262.7 |
| ML-26 | 37.108 | -122.171 | coastal | ocean-facing | whisker | M | adult | 1592.1 |
| ML-27 | 37.108 | -122.171 | coastal | ocean-facing | whisker | M | adult | 1079.7 |
| ML-28 | 37.096 | -122.205 | coastal | ocean-facing | whisker | M | adult | 1299.3 |
| ML-29 | 37.096 | -122.205 | coastal | ocean-facing | whisker | M | adult | 896.7 |
| ML-30 | 37.154 | -122.208 | coastal | ocean-facing | whisker | M | adult | 2305.8 |
| ML-31 | 37.154 | -122.208 | coastal | ocean-facing | whisker | M | adult | 237.9 |
| ML-32 | 37.098 | -122.198 | coastal | ocean-facing | whisker | F | adult | 951.6 |
| ML-33 | 37.098 | -122.198 | coastal | ocean-facing | whisker | F | adult | 567.3 |
| ML-34 | 37.029 | -122.179 | coastal | ocean-facing | whisker | F | adult | 1701.9 |
| ML-35 | 37.029 | -122.179 | coastal | ocean-facing | whisker | F | adult | 878.4 |
| ML-36 |  |  | coastal |  | whisker |  | adult | 2067.9 |
| n-01 | 36.996 | -121.716 | coastal | ocean-facing | whisker/fur | M | adult | 549 |
| n-02 | 37.02 | -122.036 | coastal | ocean-facing |  | F | adult | 1267.9 |
| n-03 | 37.112 | -121.927 | coastal | ocean-facing | whisker/fur | F | adult | 334.5 |
| n-04 | 37.087 | -121.963 | coastal | ocean-facing | whisker/fur | M | adult | 511.1 |
| n-05 | 36.53 | -121.737 | coastal |  | whisker/fur | F | adult | 2038.8 |
| n-06 | 37.14 | -122.006 | coastal |  | whisker/fur |  | sub adult | 452.4 |
| n-07 |  |  | coastal |  | whisker |  | adult | 774.4 |
| n-08 | 37.075 | -121.674 | coastal | bay-facing | whisker/fur |  | adult | 145.9 |
| n-09 | 37.112 | -121.927 | coastal |  | whisker/fur | F | sub adult | 239.7 |
| n-10 | 37.02 | -122.036 | coastal | ocean-facing | whisker | F | adult | 1279.4 |
| n-11 | 36.543 | -121.891 | coastal |  | whisker | M | adult | 2323.7 |
| n-12 | 37.468 | -122.292 | coastal | bay-facing | whisker/fur | F | adult | 3344.1 |
| n-13 | 37.139 | -122.067 | coastal | ocean-facing | whisker | M | adult | 632.2 |
| n-14 | 37.205 | -121.992 | coastal | bay-facing | whisker/fur | F | adult | 893.9 |
| n-15* | 37.063 | -122.253 | coastal |  | whisker/fur |  | sub adult | 3092.2 |
| n-16 | 36.471 | -121.841 | coastal |  | whisker/fur | M | adult | 1653.8 |
| n-17 | 37.215 | -121.988 | coastal | bay-facing | whisker/fur | M | adult | 742.5 |
| n-18 | 37.106 | -121.975 | coastal | ocean-facing | whisker/fur | M | adult | 729 |
| n-19 | 36.57 | -121.912 | coastal |  | whisker/fur | F | adult | 8078.6 |
| n-20 | 37.353 | -122.108 | coastal |  | whisker/fur | F | sub adult | 495.2 |
| n-21 | 37.285 | -122.079 | coastal |  | whisker/fur | F | sub adult | 609.8 |
| n-22 | 36.529 | -121.919 | coastal |  | whisker/fur | F | sub adult | 1374.8 |
| n-23 | 37.006 | -121.868 | coastal | ocean-facing | whisker/fur | M | adult | 397.2 |
| n-24 | 37.383 | -121.8 | coastal | bay-facing | whisker/fur | F | adult | 215.5 |
| n-25 | 37.141 | -122.114 | coastal | ocean-facing | whisker/fur | M | adult | 334.5 |
| n-26 | 37.207 | -121.987 | coastal | bay-facing | whisker | M | adult | 2254.3 |
| n-27 | 37.014 | -122.014 | coastal | ocean-facing | whisker/fur | M | adult | 2167.1 |
| n-28 | 37.004 | -121.708 | coastal | ocean-facing | whisker/fur | M | adult | 658.5 |
| n-29 | 37.2 | -121.993 | coastal | bay-facing | whisker |  | adult | 856 |
| n-30 | 37.597 | -122.42 | coastal | bay-facing | whisker/fur | M | adult | 867.1 |
| n-31 | 37.009 | -122.164 | coastal | ocean-facing | whisker | F | adult | 2667.3 |
| n-32 | 37.079 | -121.676 | coastal | bay-facing | whisker/fur | M | adult | 816.7 |
| n-33 | 36.53 | -121.737 | coastal |  | whisker/fur | F | adult | 806.4 |
| n-34 | 37.017 | -122.187 | coastal | ocean-facing | whisker/fur | M | adult | 2159.3 |
| n-36 | 37.009 | -122.164 | coastal | ocean-facing | whisker | M | adult | 1368 |
| n-37 | 37.139 | -122.066 | coastal | ocean-facing | whisker | M | adult | 822.2 |
| n-38 | 37.081 | -122.158 | coastal | ocean-facing | whisker/fur | F | adult | 3576.5 |
| n-39 | 36.977 | -122.018 | coastal | ocean-facing | whisker/fur | M | adult | 644.3 |
| n-40 | 37.175 | -121.979 | coastal |  | whisker/fur | F | kitten | 384.6 |
| n-41 | 37.094 | -122.2 | coastal |  | whisker/fur | M | sub adult | 763.3 |
| n-42 | 37.077 | -122.186 | coastal | ocean-facing | whisker/fur | M | adult | 1284.6 |
| n-43 | 37.239 | -122.211 | coastal | ocean-facing | whisker/fur | F | adult | 3866.6 |
| n-44 | 37.475 | -122.298 | coastal | bay-facing | whisker/fur | M | adult | 741.3 |
| n-45 | 37.101 | -122.167 | coastal | ocean-facing | whisker/fur | M | adult | 1312.3 |

Table S-4: Summary of biotic samples taken from both coastal and inland regions of California, USA.

| Species Type | Species | Tissue Types | Region | Years of Sample Collection | Sample Provider | Sample Collection Description |
| --- | --- | --- | --- | --- | --- | --- |
| Puma | *Puma concolor* | Fur and whisker | Coastal | 2008-2014 | UCSC | Collected during necropsy and tracking of wild animals |
|  |  | Whisker | Inland | 2006-2010 | CDFW | Collected during necropsy |
|  |  | Fur | Inland | 1916-1933 | UC Berkeley, MVZ | Collected from museum archived pelts |
| Deer | *Odocoileus hemionus* | Fur | Coastal | 2010-2012 | UCSC | Collected at puma kill sites |
|  |  | Fur | Inland | 2008-2012 | CDFW | Collected at hunter check stations |
| Lichen | *Ramalina menziesii* | Whole specimen | Coastal (Ocean- and bay-facing) | 2017 | UCSC | Collected from tree understory |
